# Popularity of Different Lampyrid Species in Japanese Culture as Measured by Google Search Volume

**DOI:** 10.3390/insects2030336

**Published:** 2011-07-05

**Authors:** Kenta Takada

**Affiliations:** 3-13-29, Takejima, Nishiyodogawa-ku, Osaka 555-0011, Japan; E-Mail: athemus99@yahoo.co.jp; Tel.: +81-6-6473-4128; Fax: +81-6-6473-4128

**Keywords:** popularity, lampyrids, Japanese culture, cultural entomology

## Abstract

I investigated the popularity of different lampyrid species (34 species) in Japanese culture as part of a study on cultural entomology. Popularity was assessed by the Google search volume for Japanese lampyrid species names in katakana and hiragana scripts, using the Keyword Tool of Google AdWords. The search volume of lampyrid species as “Genji-botaru” (*Luciola cruciata* Motschulsky), “Heike-botaru” (*Luciola lateralis* Motschulsky) and “Hime-botaru” (*Hotaria parvula* Kiesenwetter), in either or both katakana and hiragana syllabic scripts, was enormously high relative to other lampyrid species, indicating the biased attention of Japanese to these lampyrid species. In addition, search volumes for familial or common lampyrid name (“Hotaru”) was assessed and compared with that of 34 lampyrid species. This analyzing result showed that: (1) the search volumes for katakana and hiragana were 37.7 and 773.1 times higher for “Hotaru” than “Genji-botaru”, respectively; and (2) the search volume for all lampyrid species was clearly higher in katakana than hiragana, whereas the search volumes for “Hotaru” were clearly higher in hiragana than katakana. These results suggest that: (1) the Japanese public tends to perceive lampyrids with not a clear but an ambiguous taxonomic view; and (2) the attitude of the Japanese public toward lampyrids differs between those who perceive lampyrids with a clear taxonomic view (at species level) and with an ambiguous taxonomic view.

## 1. Introduction

The field of cultural entomology examines the influence of insects on human societies [1–5]. A fascinating question in cultural entomology is which and how insect groups are represented in human culture, *i.e.*, the popularity of insects in human societies [3,4,6,7]. Precisely, the popularity of different insect groups is an indicator of their influence on human societies.

Previous studies on cultural entomology mentioned the influence of various insects on human practice with biased attention of humans to a small number of insect groups [6–10]. For example, Coelho [6] shows that a small number of insect orders, Hymenoptera, Lepidoptera and Diptera, appear frequently in western popular music of the rock and roll era. Also, Takada [4] revealed that “Hotaru” (Lampyrids) and “Kabuto-mushi” (Dinastines or Japanese rhinoceros beetles) are extraordinarily popular coleopteran groups in Japanese culture and suggested that the high popularity of some coleopteran groups is due to their apparent characteristics to humans; however, these studies focused on the popularity of different insect orders and families. Thus, questions remain as to which and how lesser taxonomic groups of insect orders and families as genus or species are represented in our culture.

Lampyridae is the most attractive coleopteran family in the field of cultural entomology, because lampyrids influence various aspects of human practice and, especially in Japan, they have assumed a position of unique cultural significance due to their bioluminescence [4,5,11–14]. Although It has been mentioned that only a small number of lampyrid species, such as the Genji-firefly (*Luciola cruciata* Motschulsky), Heike-firefly (*Luciola lateralis* Motschulsky) and Hime-firefly (*Hotaria parvula* Kiesenwetter), which have the characteristics of conspicuous bioluminescence and their occurrence around human habitation, are extraordinarily popular Japanese lampyrids and are perceived as a common type of lampyrids in Japanese culture [13,14], there has been no numerical analysis of the popularity of different lampyrid species. Such analyses of their popularities will provide much insight to understand the Japanese appreciation and view toward conservation of insects and nature, because lampyrids have been widely accepted as an excellent symbol of nature and its conservation by Japanese public [13].

I therefore investigated the popularity of different lampyrid species and examined which and how lampyrid species are represented in Japanese culture. The Japanese have a highly developed tradition of aesthetic appreciation of insects and use them in various cultural contexts [1–3,5,6,15–17], indicating the importance of Japanese culture in light of cultural entomology. The popularity of different lampyrid species was assessed by the Google search volume of group names. This statistic is used as a yardstick to measure a term's intention, interest or popularity, and is thus applied for internet marketing and search engine optimization [18,19]. This statistic also breaks out of methodological constraints, which have limited the cultural entomologist's attempt to investigate the popularity of different insect groups [4].

## 2. Methods

I conducted a survey on the popularity of lampyrid species on 19 February 2011, assessing the global monthly search volume using the Keyword Tool in Google AdWords [20]. The global monthly search volume shows the approximate average monthly number of search queries matching each keyword result. This statistic (called “search volume”) applies to searches performed on Google and the search network over the past 12-month period. When Google AdWords has insufficient data on a particular keyword, it returns “–” (not enough data). Such a case is regarded as having no search volume (0) for the keyword.

**Figure 1:**
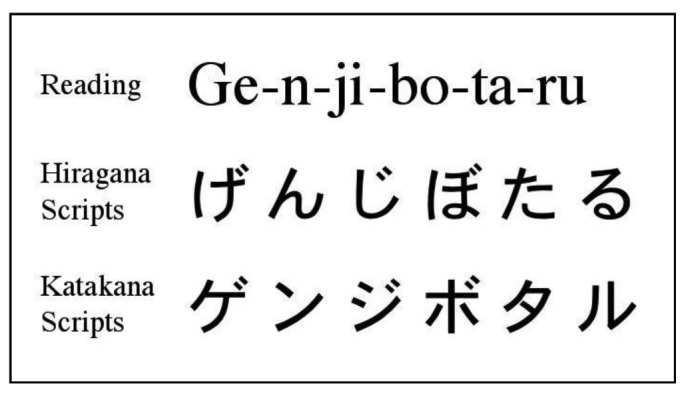
Japanese transcription (Hiragana and Katakana) for the “Genji-botaru” (Genji-firefly, *Luciola cruciata* Motschulsky).

I used 34 species names of lampyrids as keywords to evaluate the search volume of lampyrid species. I referred to the Japanese names of these lampyrid species in Kurosawa *et al.* [21] because this reference is one of the most popular and famous illustrated books on coleopteran insects in Japan. The search volume of Japanese names of these lampyrid species was assessed in hiragana and katakana, which are Japanese syllabic scripts, components of the Japanese writing system (Figure 1). Hiragana is used for words for which there are no kanji, and in words for which the kanji form is not known to the writer or readers, or is too formal when written. Katakana is most often used for the transcription of words from foreign languages, onomatopoeia and technical and scientific terms, such as the names of animal and plant species and minerals [4]. In addition to 34 species names of lampyrids, I also examined the search volume of “Hotaru”, which is the familial and common lampyrid name.

To evaluate the search volume, I employed the browser Internet Explorer 8.0. The operating system was Windows Vista Home Premium Service Pack 2 installed on a PC-LL800KG/Lavie L (CPU: Intel Core2 Duo T7250 (2.0 GHz)).

## 3. Results and Discussion

The search volume for “Genji-botaru”, which is the Genji-firefly (*Luciola cruciata*) in Japanese, was 2400 searches, the highest of the species names of lampyrid in katakana (Table 1). The search volume for “Hime-botaru” (*Hotaria parvula*) was the second highest in katakana (1300 searches), and the search volume for “Heike-botaru” (*Luciola lateralis*) was the third highest in katakana (1000 searches). For species names in katakana, a search volume frequency of 100 to less than 1000 searches and of 10 to less than 100 searches occurred for two and five species, respectively. On the other hand, no search volume was obtained for 24 species names in katakana, due to the lack of data on these keywords in Google AdWords. Of the species names of lampyrid in hiragana, only the search volume for the species “Genji-botaru” (*Luciola cruciata*) was over 100 searches (260 searches) and no search volume was obtained for a further 33 species names in hiragana. As a whole, the search volume of all lampyrid species was higher in katakana than hiragana.

The search volumes for “Genji-botaru” (*Luciola cruciata*), “Hime-botaru” (*Hotaria parvula*) and “Heike-botaru” (*Luciola lateralis*) in either or both katakana and hiragana scripts were enormously high relative to other lampyrid species (Table 1), indicating that these species are extraordinarily popular in Japanese culture, as mentioned by several studies [13,14]. In general, Japanese people perceive these species as common types of lampyrids and, historically, chasing these lampyrid species for their bioluminescence (*Hotaru-gari*) has been a traditional pastime that gives some poetic charm to early summer evenings in Japan [3–5,11–14,22–26].

As a whole, a relatively small number of lampyrid species was represented by an extraordinarily high search volume, while an abundance of other lampyrid species was represented by a low search volume, indicating the biased attention of Japanese to only a small number of lampyrid species (Table 1). It appears that the most popular lampyrids have characteristics of apparent biological traits, such as conspicuous bioluminescence by enormous swarms of *Luciola cruciata*. In addition, these species occur around human habitation where people can view or chase fireflies safely because of the absence of dangerous animals. On the other hand, most lampyrid species have characteristics of less apparent biological traits, such as weak or non-bioluminescence, nonswarming habit and/or occurrence far from human habitation, and thus are perhaps not found and perceived by casual observers [13]. These explanations are supported by Takada [4,5], who suggested that most popular coleopterans have characteristics of: (1) apparent morphological and ecological traits; (2) association with human survival (beneficial insects and pests); and/or (3) occurrence around human habitation.

In addition to 34 species names of lampyrids, I also examined the search volume of “Hotaru”, which is the familial and common lampyrid name. As a result, the search volumes for “Hotaru” were 90,500 in katakana and 201,000 in hiragana, 37.7 times and 773.1 times the search volumes for “Genji-botaru” (Genji-fireflies) in katakana and hiragana, respectively. This result suggests that the majority of the Japanese general public tends to perceive lampyrids not with a clear taxonomic view but with an ambiguous taxonomic view, although they pay extraordinary attention to lampyrids [4,5]. However, although “Hotaru” is the familial and common lampyrid name, which symbolizes the ambiguous taxonomic view of Japanese for fireflies, “Hotaru” usually refers to the Genji-fireflies (*Luciola cruciata*) in Japan [13].

Moreover, the search volume for all lampyrid species was clearly higher in katakana than hiragana, whereas the search volumes of “Hotaru” were clearly higher in hiragana than katakana, as mentioned by Takada [4]. These results suggest that the attitude of the Japanese public toward lampyrids differs between those who perceive lampyrids with a clear taxonomic view and with an ambiguous taxonomic view, because Japanese syllabic scripts (hiragana and katakana) are chosen according to the writing purpose [4]. These results weakly suggest that lampyrid species names are directly used for biological or biologically related interests as compared with the familial and common lampyrid name, which is often used symbolically or casually for non-biological interests.

## 4. Conclusions

Japanese general public pay extraordinary attention to a small number of lampyrid species, such as “Genji-botaru” (*Luciola cruciata* Motschulsky), “Heike-botaru” (*Luciola lateralis* Motschulsky) and “Hime-botaru” (*Hotaria parvula* Kiesenwetter), which have the characteristics of conspicuous bioluminescence and their occurrence around human habitation. In addition, Japanese general public tends to perceive lampyrids not with a clear taxonomic view but with an ambiguous taxonomic view and perhaps appreciate lampyrids casually with non-biological interest, although they pay extraordinary attention to lampyrids

These findings will contribute to understanding the Japanese attitude toward insects and nature in general. Perhaps these findings suggest that Japanese appreciation and view toward conservation for nature was found to be very narrow and idealized, primarily focusing on particular species and lacking a biological and ecological perspective according to their particular aesthetic value and casual view, as mentioned by Kellert [27]. Japanese appreciation and view toward conservation for nature in associated with lampyrids may be biased to a narrow viewpoint, due to the extraordinary attention paid to a small number of species, although lampyrids have been widely accepted as an excellent symbol of nature and its conservation by the Japanese general public.

## Figures and Tables

**Table 1:** Google search volume for different lampyrid species (32 species) and common (or familial) lampyrid name.

**Subfamily**	**Species (Latin)**				**Species (Japanese)**	**Search Volume**	

						**Katakana**	**Hiragana**
Psilocladinae	*Cyphonocerus*	*ruficollis*	Kiesenwetter	1879	[Mune-kuriiro-botaru]	36	0
		*marginatus*	Lewis	1895	[Heriaka-kushihige-botaru]	0	0
		*yayeyamensis*	M. Sato	1976	[Yaeyama-kushihige-botaru]	0	0
Ototretinae	*Drilaster*	*axillaris*	Kiesenwetter	1879	[Katamon-minami-botaru]	0	0
		*unicolor*	Lewis	1895	[Kuro-minami-botaru]	0	0
		*shibatai*	M. Sato	1968	[Amami-minami-botaru]	0	0
		*okinawensis*	Nakane	1977	[Okinawa-kuro-minami-botaru]	0	0
		*bicolor*	M. Sato	1968	[Aka-mimami-botaru]	0	0
		*fuscicollis*	Nakane	1977	[Okinawa-aka-minami-botaru]	0	0
		*ohbayashii*	M. Sato	1968	[Oobayash-minami-botaru]	0	0
	*Stenocladius*	*bicoloripes*	Pic	1918	[Kiiro-husahige-botaru]	0	0
		*azumai*	Nakane	1981	[Tateobi-husahige-botaru]	0	0
		*shirakii*	Nakane	1981	[Kiberi-husahige-botaru]	0	0
Luciolinae	*Luciola*	*kuroiwae*	Matsumura	1918	[Kuroiwa-botaru]	0	0
		*filiformis*	Matsumura	1918	[Yaeyama-hime-botaru]	0	0
		*cruciata*	Motschulsky	1854	[Genji-botaru]	2400	260
		*lateralis*	Motschulsky	1860	[Heike-botaru]	1,000	0
	*Hotaria*	*parvula*	Kiesenwetter	1874	[Hime-botaru]	1300	0
		*papariensis*	Doi	1932	[Papari-botaru]	0	0
	*Curtos*	*costipennis*	Gorham	1880	[Kiiro-suji-botaru]	0	0
		*okinawanus*	Matsumura	1918	[Okinawa-suji-botaru]	0	0
Lampyrinae	*Lychnuris*	*fumosa*	Gorham	1883	[Kuro-mado-botaru]	140	0
		*discicollis*	Kiesenwetter	1874	[Oo-mado-botaru]	22	0
		*miyako*	Nakane	1981	[Miyako-mado-botaru]	0	0
		*matsumurai*	Nakane	1963	[Okinawa-mado-botaru]	0	0
		*atripennis*	Lewis	1896	[Ooshima-mado-botaru]	0	0
		*rufa*	Olivier	1886	[Aki-mado-botaru]	16	0
		*abdominalis*	Nakane	1977	[Sakishima-mado-botaru]	0	0
	*Lucidina*	*accensa*	Gorham	1883	[Oo-oba-botaru]	22	0
		*biplagiata*	Motschulsky	1866	[Oba-botaru]	110	0
		*natsumiae*	Chujo et M. Sato	1972	[Natsumi-oba-botaru]	0	0
		*okadai*	Nakane et Ohbayashi	1949	[Kokuro-oba-botaru]	0	0
	*Pristolycus*	*sagulatus*	Gorham	1883	[Sujiguro-botaru]	36	0
		*shikokensis*	Ohbayashi et M. Sato	1963	[Shikoku-sujiguro-botaru]	0	0
	Lampirid familial or common name				[Hotaru]	90,500	201,000
